# Glucocorticoid boluses followed by tocilizumab in giant cell arteritis patients: effects on peripheral blood monocytes and lymphocytes

**DOI:** 10.3389/fimmu.2025.1516008

**Published:** 2025-03-27

**Authors:** Cecilia Catellani, Martina Bonacini, Alessandro Rossi, Ilaria Ferrigno, Veronica Buia, Chiara Marvisi, Giulia Cassone, Mariagrazia Catanoso, Gabriella Di Tommaso, Luigi Boiardi, Rexhep Durmo, Annibale Versari, Massimiliano Casali, Giulia Besutti, Lucia Spaggiari, Alessandro Zerbini, Francesco Muratore, Carlo Salvarani, Stefania Croci

**Affiliations:** ^1^ Unit of Clinical Immunology, Allergy and Advanced Biotechnologies, Azienda Unità Sanitaria Locale-IRCCS di Reggio Emilia, Reggio Emilia, Italy; ^2^ PhD Program in Clinical and Experimental Medicine, University of Modena and Reggio Emilia, Modena, Italy; ^3^ Unit of Rheumatology, Azienda Unità Sanitaria Locale-IRCCS di Reggio Emilia, Reggio Emilia, Italy; ^4^ Unit of Nuclear Medicine, Azienda Unità Sanitaria Locale-IRCCS di Reggio Emilia, Reggio Emilia, Italy; ^5^ Nuclear Medicine Unit, Azienda Unità Sanitaria Locale di Piacenza, Piacenza, Italy; ^6^ Department of Surgery, Medicine, Dentistry and Morphological Sciences with Interest in Transplant, Oncology and Regenerative Medicine, University of Modena and Reggio Emilia, Modena, Italy; ^7^ Unit of Radiology, Azienda Unità Sanitaria Locale-IRCCS di Reggio Emilia, Reggio Emilia, Italy

**Keywords:** giant cell arteritis, glucocorticoids, tocilizumab, lymphocytes, monocytes, CCR2

## Abstract

**Introduction:**

Giant Cell Arteritis (GCA) is the most common vasculitis in the elderly, characterized by granulomatous infiltration of immune cells in medium and large arteries. A therapeutic protocol that combines ultra-short glucocorticoids (GC) followed by tocilizumab (TCZ) monotherapy has been proven effective in GCA patients with extracranial large vessel involvement (LV-GCA). However, its effects on circulating immune cells are unknown. The aim of this study was to deepen the understanding of the immunological mechanisms behind this treatment regimen in patients with LV-GCA.

**Methods:**

15 patients with active LV-GCA were included in this study. Blood samples were collected at baseline, after 3 days of GC treatment, at weeks 24 and 52 during TCZ monotherapy, and at week 78 after the suspension of TCZ. Peripheral blood mononuclear cells were isolated from blood samples. The percentages of lymphocyte and monocyte subsets and the expression of the monocyte markers CCR2, CX3CR1, and HLA-DR were analyzed by flow cytometry. Paired Student’s t-test and mixed-effects analysis were used for the comparison between and among groups, respectively.

**Results:**

GC boluses increased the percentages of B lymphocytes and classical monocytes while decreased those of CD4+ T lymphocytes and intermediate and non-classical monocytes. Moreover, GC boluses increased CCR2 and decreased HLA-DR and CX3CR1 expression by monocytes. TCZ induced a reduction in CCR2 expression versus baseline in classical and intermediate monocytes. Patients with higher reduction in CCR2 expression in intermediate monocytes at 24 weeks and 52 weeks versus baseline showed signs of disease activity at 78 weeks.

**Conclusion:**

GC boluses modified the relative percentages of lymphocyte and monocyte subsets and modified the expression levels of CCR2, CX3CR1, and HLA-DR in monocytes. These changes may contribute to the anti-inflammatory effects of GCs. TCZ monotherapy had more limited effects. Changes in CCR2 expression by intermediate monocytes might have a prognostic value in LVV.

## 1 Introduction

Large vessel vasculitis (LVV) is a pathological condition characterized by the inflammation of the aorta and its major branches, which includes giant cell arteritis (GCA) and Takayasu arteritis based on the pattern of arterial involvement, age of patients and clinical characteristics (1). GCA is the most common vasculitis in adults having more than 50 years (2).

Cranial arteries (cranial GCA, C-GCA) can be predominantly affected, leading to cranial symptoms. In other patients, the aorta and its branches (termed large vessel GCA, LV-GCA) are primarily involved, typically without cranial manifestations. In C-GCA, the most feared complication is early visual loss, while in LV-GCA, aortic aneurysms or stroke are the main fears (or concerns), generally occurring as late complications (3).

To date, glucocorticoids (GC) are the first therapeutic choice for the management of GCA (4) but their long-term administration is associated with severe side effects (5). Monotherapy with tocilizumab (TCZ), an IL-6 receptor inhibitor, without associated GCs has been reported to be effective in improving clinical symptoms and systemic markers of inflammation in a small group of newly diagnosed LVV patients (6). Moreover, TCZ has been reported to effectively reduce disease flares when used with high-dose GCs and have a GC-sparing effect (7, 8). Recent data suggest that TCZ monotherapy, administered after ultra-short-pulse GC therapy, can control clinical symptoms of GCA, reduce vascular inflammation, and maintain remission in about 50% of patients with GCA (3, 9–11).

However, the mechanisms of action of this therapeutic protocol in LV-GCA remain poorly understood. Current data from serum proteomics indicate that disease-associated serum proteins improve with treatment, highlighting the differing effects of the two drugs (12, 13).

This study aimed to evaluate the effects of a treatment regimen consisting of ultra-short pulses of GCs followed by TCZ monotherapy on circulating lymphocyte and monocyte subsets, as well as monocyte markers in patients with LV-GCA. In particular, the primary aim was to assess the effects of TCZ monotherapy on lymphocytes and monocytes at 24 and 52 weeks compared to baseline. Secondary objectives included evaluating predictive biomarkers of disease activity after TCZ discontinuation (78 weeks *versus* baseline) and the effects of GC pulses (after the 3^rd^ dose *versus* baseline). We focused on the main lymphocyte subsets known to have a role in LV-GCA (i.g. CD4+ T lymphocytes) and classical, intermediate and non-classical monocytes expressing CCR2, CX3CR1 known to be deregulated in LV-GCA. The expression of HLA-DR by monocytes was chosen to evaluate their antigen presentation potential. Understanding the mechanisms of action of TCZ and GC pulse therapy can help identify key mediators, for developing new targeted therapies. Additionally, stratifying patients into responders and non-responders after TCZ discontinuation may reveal potential markers for predicting TCZ response in LV-GCA.

## 2 Materials and methods

### 2.1 Patients

Fifteen patients with newly diagnosed or relapsing LV-GCA enrolled at the Unit of Rheumatology, Azienda USL-IRCCS di Reggio Emilia, Italy in the prospective observational study: “Treatment Of giant cell arteritis Patients with ultra-short glucocorticoids And tociliZumab: the role of Imaging in a prospective Observational study (TOPAZIO, ClinicalTrials.gov ID: NCT05394909)” (3) were included in the present study. LV-GCA was defined by the presence of large-vessel inflammation on PET/CT, with or without cranial manifestations and pathological or ultrasonography evidence of temporal artery involvement. In particular, the inclusion criteria were: age ≥ 50 years; PET/CT evidencing a FDG uptake with visual score ≥ 2 (equal or above liver uptake) in at least one large artery considered consistent with active vasculitis by the evaluation of a nuclear medicine physician with long-term expertise in vasculitis; at least one of: ESR > 40 mm/h or CRP >10 mg/l; cranial or systemic symptoms of GCA or polymyalgia rheumatica (PMR). The exclusion criteria were: history or presence of GCA-related ischemic cranial manifestations; treatment with > 10 mg/day of prednisone (or equivalent) for >10 consecutive days in the previous 3 months; previous treatment with TCZ. Clinical characteristics of patients at baseline are reported in **Supplementary Table S1**.

Patients were treated with 500 mg of intravenous methylprednisolone on days 1, 2 and 3 in 250 ml saline solution. Thereafter, GC treatment was suspended and patients received weekly subcutaneous injections of 162 mg of TCZ from day 4 until week 52. Disease assessment was conducted during each visit at days 1, 4 and 31 and every 12 weeks thereafter (3). Patients in clinical remission at week 52 stopped TCZ and entered in a 26-week observational period, until week 78 (9).

At 78 weeks, patients were classified as responders to treatment if they met all of the following remission criteria: absence of any clinical signs and symptoms directly attributable to GCA; normalization of CRP and ESR values; absence of new or worsened aortic damage at CT; vascular FDG uptake < 2 in all large arteries at PET/CT or overall PET image interpretation of non-active vasculitis by the nuclear medicine physician. Patients who did not meet these criteria were classified as non-responders to treatment.

The study protocol was approved by the Reggio Emilia Ethics Committee Area Vasta Emilia Nord (0176 - 15/05/2019) and registered in ClinicalTrials.gov (NCT05394909). All patients provided written informed consent before enrolment. The study was conducted in accordance with the Declaration of Helsinki.

### 2.2 Biological sample collection and PBMC isolation

Blood samples were collected into EDTA-coated tubes at baseline (T0), after 3 days of GC administration (post-GC), at 24 and 52 weeks of TCZ monotherapy, and at week 78 of follow-up, coinciding with PET/CT evaluations. These time points were selected to obtain biological samples at the start of treatment and TCZ monotherapy, and subsequently to correlate the immunological data with vascular disease activity as measured by PET/CT imaging. Peripheral blood mononuclear cells (PBMCs) were isolated by Histopaque-1077 density gradient centrifugation (Sigma-Aldrich) and stored frozen in liquid nitrogen in 90% heat-inactivated fetal bovine serum (FBS, Gibco, ThermoFisher) 10% dimethyl sulfoxide (DMSO, Sigma-Aldrich) until use. The use of post-thawed PBMCs allowed to analyze all the longitudinal samples from each patient in the same assay reducing technical variability while increasing the strength of the comparisons.

### 2.3 Flow cytometry

PBMCs were thawed and counted in Trypan Blue and Turk’s solutions. For each patient both lymphocyte and monocyte subsets were analyzed by flow cytometry. 200.000 PBMCs from each patient at each time point were suspended in 100 μL Phosphate-Buffered Saline (PBS, Gibco, ThermoFisher) + 0.1% LIVE/DEAD Fixable Near IR stain (L/D, Invitrogen, L10119) and incubated for 10 min at room temperature. After washing with PBS + 1% FBS, to define lymphocyte subsets, PBMCs were suspended in 100 µl of PBS + 1% FBS containing the following antibodies: 0.5 µl PerCP mouse anti-human CD3 (Miltenyi, clone BW264156), 0.5 µl PE anti-human CD56 (Miltenyi, clone REA196), 1 µl FITC anti-human CD8 (Miltenyi, clone REA734), 0.5 µl PE-Vio770 anti-human CD19 (Miltenyi, clone REA675), and 0.5 µl APC anti-human CD4 (Miltenyi, clone REA623). To define the monocyte subsets and to analyze the expression of monocyte markers, PBMCs were suspended in 100 µl of PBS + 1% FBS containing the following antibodies: 2 µl PerCP-Vio 770 anti-human HLA-DR (Miltenyi, clone REA805), 2.5 µl PE anti-human CX3CR1 (Miltenyi, clone REA385), 10 µl PE-Vio770 anti-human CCR2 (Miltenyi, clone REA624), 10 µl APC anti-human CD14 (BD Biosciences, clone M5E2), 5 µl FITC anti-human CD16 (BD Biosciences, clone 3G8). Quantities of antibodies were defined by titration assays. Cells were stained for 30 min at 4°C. After washing, PBMCs were suspended in PBS + 1% FBS and acquired with the FACS Canto II flow cytometer (BD Biosciences), equipped with two lasers for excitation at 488 nm and 633 nm. At least 10.000 lymphocytes or 3.000 monocytes were acquired in each sample based on the forward and side scatter of the cells. Only viable cells were analyzed based on the cell viability fluorescent dye. Data were analyzed with Kaluza software 2.1 (Beckman Coulter). Gates were defined using fluorescence minus one (FMO) controls. Gating strategy is shown in **Supplementary Figures S1, S2**.

### 2.4 Statistical analysis

Statistical analysis was performed using GraphPad Prism 10 software. Paired Student’s t-test was used to analyze the effects of GCs on the percentages of lymphocyte subsets, monocyte subsets, and on the expression of monocyte markers as mean fluorescence intensity (MFI) with respect to the baseline. Mixed-effects analysis with Tukey’s test correction for multiple comparisons was used to evaluate the effects of TCZ on the percentages of lymphocyte subsets, monocyte subsets and on the expression of monocyte markers among the different time points on TCZ monotherapy. Student’s t-test was used to investigate the differences in the monocyte marker MFIs, fold changes in these MFI, percentages of lymphocyte and monocyte subsets, and increase/decrease in such percentages at 24 weeks and 52 weeks between responder and non-responder patients. Receiver Operating Characteristic (ROC) curve analysis was applied to determine cut-off values to discriminate between responders and non-responders. P-values <0.05 (two-tailed) were considered statistically significant.

## 3 Results

Blood samples were collected from all patients at baseline and post-GC (n = 15), from 12 patients up to week 24, from 10 patients up to week 52, and from 9 patients up to week 78.

### 3.1 Effects of GC boluses on lymphocyte subsets

**Figure 1:**
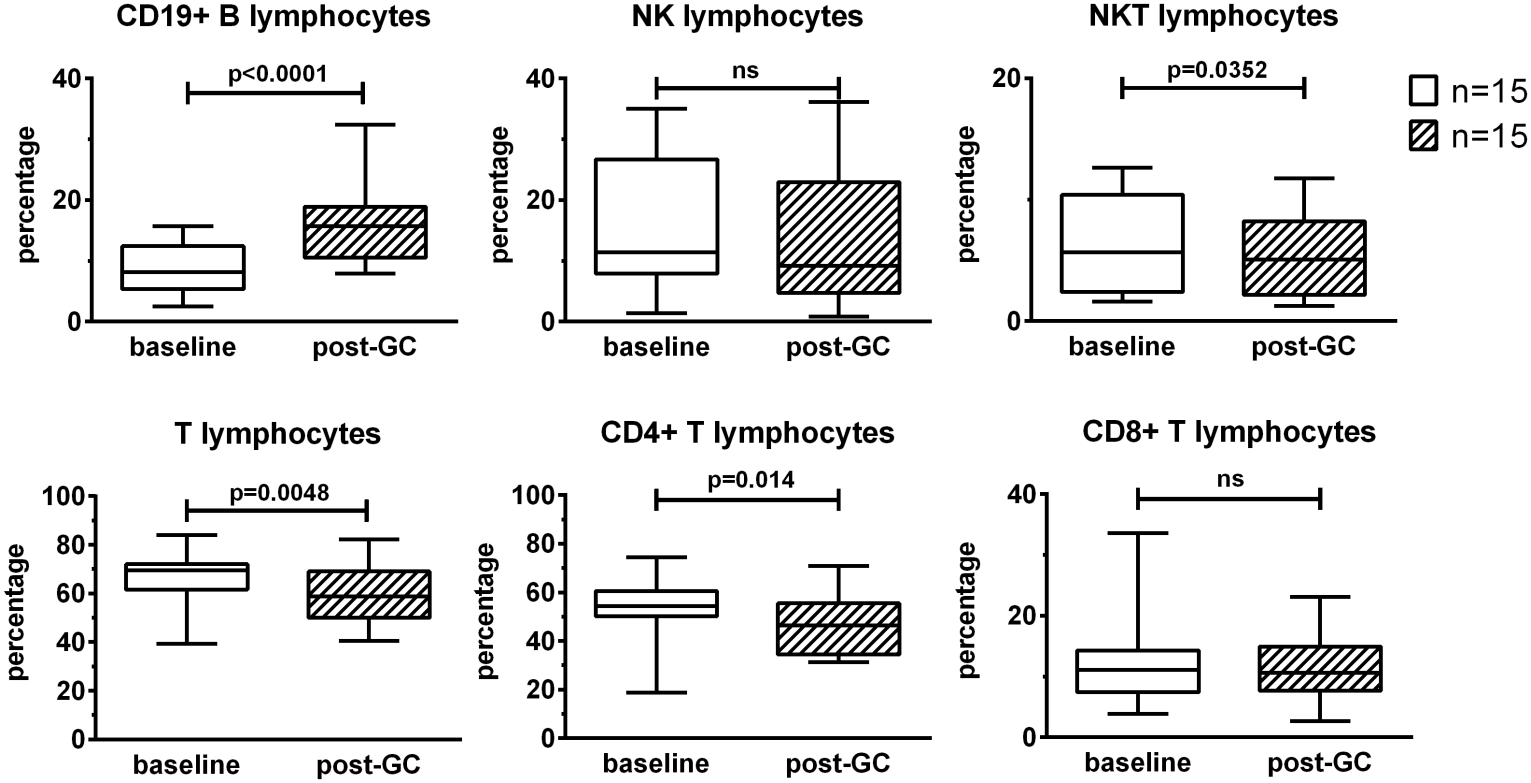
Effects of GC boluses on lymphocyte subsets in patients with LV-GCA. Lymphocyte subsets were identified by means of flow cytometry using the following surface markers: CD3, CD4, CD8, CD19, and CD56. Percentages were calculated in the lymphocyte gate defined on forward and side scatter of the cells. The data before (baseline) and after 3 days of GC treatment (post-GC) were compared by paired Student’s t-test (n=15). P-values <0.05 were considered statistically significant. Box plots represent the median ± interquartile range. The whiskers represent the highest and the lowest values. ns, not significant.

To evaluate the effects of GC treatment on lymphocyte subsets, PBMCs were analyzed by flow-cytometry after 3 boluses of GCs compared to baseline. GCs induced an increase in the percentages of CD19+ B lymphocytes (+ 8%), a decrease in the percentages of T lymphocytes (-7.4%) and particularly CD4+ T lymphocytes (- 6.3%) and a slight decrease in the percentages of NKT lymphocytes (- 1%). The percentages of NK lymphocytes and CD8+ T lymphocytes were not impacted by GC therapy (**Figure 1**).

### 3.2 Effects of TCZ monotherapy on lymphocyte subsets over time

To evaluate the effects of treatment with boluses of GCs followed by TCZ monotherapy on lymphocyte subsets, PBMCs were analyzed longitudinally in each patient at baseline, after 3 days of GC treatment, at 24 and 52 weeks of TCZ monotherapy and at 78 weeks of follow-up. No differences were found in the percentages of B, NK, NKT, T, CD4+ T, and CD8+ T lymphocytes during and after TCZ monotherapy with respect to the baseline (**Supplementary Figure S3**). A decrease in the percentages of B lymphocytes and an increase in the percentages of NKT lymphocytes were observed at 52 weeks of TCZ monotherapy with respect to GC. Moreover, an increase in the percentages of NKT lymphocytes was observed also at 24 weeks on TCZ monotherapy with respect to GC. One patient showed high percentages of CD8+ T lymphocytes compared to the other patients during the entire period of the study.

### 3.3 Effects of GC boluses on monocyte subsets

**Figure 2:**
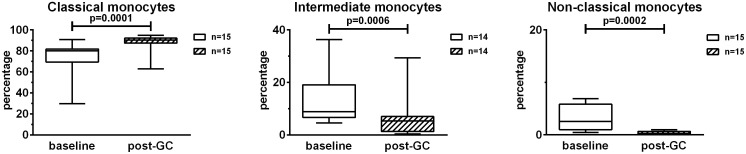
Effects of GC boluses on monocyte subsets in patients with LV-GCA. Monocyte subsets were identified in PBMCs by means of flow cytometry using anti-CD14 and anti-CD16 antibodies. Percentages were calculated in the monocyte gate. The data before (baseline) and after 3 days of GC treatment (post-GC) were analyzed by paired Student’s t-test. P-values<0.05 were considered statistically significant. Box plots represent the median ± interquartile range. The whiskers represent the highest and the lowest values.

To evaluate the effects of GCs on monocyte subsets, PBMCs were analyzed by flow cytometry using antibodies anti-CD14 and anti-CD16. This allowed the identification of classical (CD14+CD16neg), intermediate (CD14+CD16+) and non-classical (CD14lowCD16+) monocytes. The percentages of classical monocytes increased after GC treatment with respect to the baseline (+ 12.9%) (**Figure 2**). The percentages of intermediate and non-classical monocytes decreased after GC treatment (- 6.7% and – 2.7% respectively). In particular, the percentages of non-classical monocytes were strongly impacted by GCs. Concerning the analysis of intermediate monocytes, one patient was excluded due to the low, baseline percentage of intermediate monocytes (= 0.27%).

### 3.4 Effects of TCZ monotherapy on monocyte subsets over time

**Figure 3:**
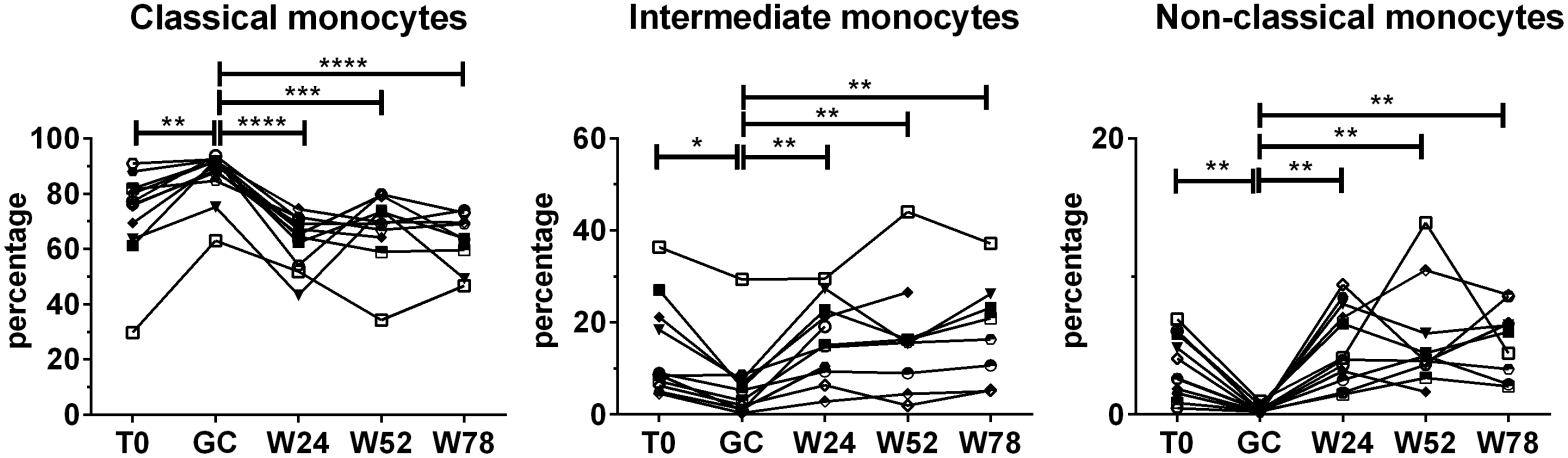
Effects of TCZ monotherapy on monocyte subsets over time. Monocyte subsets were identified in PBMCs using flow cytometry using anti-CD14 and anti-CD16 antibodies. Percentages were calculated in the monocyte gate. Data were analyzed at baseline (T0), after 3 days of GC treatment (GC), at 24 (W24) and 52 weeks (W52) of TCZ monotherapy, and at 78 weeks of follow-up (W78). Mixed-effects analysis followed by Tukey’s test correction for multiple comparisons was used to compare data among the time points. Only patients with at least one time point on TCZ treatment were considered for the analysis. Each symbol represents a different patient. P-values<0.05 were considered statistically significant. * = p<0.05; ** = p<0.01; *** = p<0.001; **** = p<0.0001..

To evaluate the effects of treatment with GCs followed by TCZ monotherapy on monocyte subsets, PBMCs were analyzed longitudinally in each patient using anti-CD14 and anti-CD16 antibodies. During TCZ monotherapy the percentages of the monocyte subsets tended to those observed at baseline and no significant differences were found in the percentages of monocyte subsets at 24 weeks, 52 weeks or 78 weeks compared with the baseline (**Figure 3**). Indeed, the observed decrease in the percentages of classical monocytes and the increase in the percentages of intermediate and non-classical monocytes during TCZ monotherapy were referred to those obtained after GC treatment. The analysis of the fold changes in the percentages of monocyte subsets on TCZ treatment compared to the baseline held similar results (**Supplementary Figure S4**).

### 3.5 Effects of GC boluses on CCR2, HLA-DR and CX3CR1 expression by classical and intermediate monocytes

**Figure 4:**
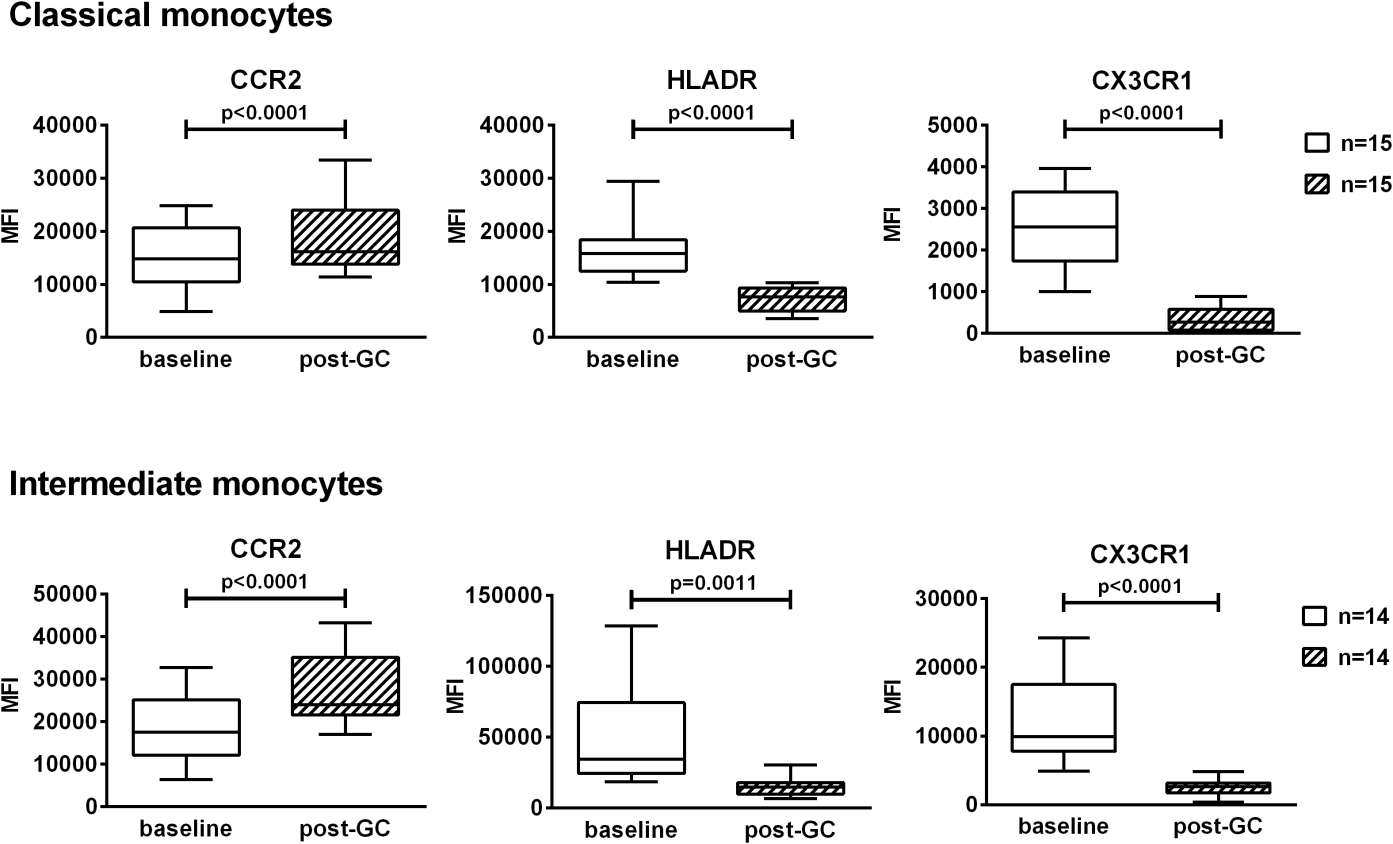
Effects of GC boluses on the expression of CCR2, HLADR and CX3CR1 in patients with LV-GCA in classical and intermediate monocytes. Monocyte subsets were identified in PBMCs by means of flow cytometry using anti-CD14 and anti-CD16 antibodies. Median fluorescence intensities (MFI) values of CCR2, HLADR, CX3CR1 are shown in classical and intermediate monocytes. The data before (baseline) and after 3 days of GC treatment (post-GC) were analyzed by paired Student’s t-test. P-values<0.05 were considered statistically significant. Box plots represent the median ± interquartile range. The whiskers represent the highest and the lowest MFI values.

The impact of GCs on the expression of the surface markers CCR2, HLA-DR and CX3CR1 was analyzed. CCR2 was expressed at high levels by classical and intermediate monocytes while the expression was low in non-classical monocytes. HLA-DR was mostly expressed by intermediate and non-classical monocytes. CX3CR1 was mostly expressed by non-classical monocytes followed by intermediate monocytes then by classical monocytes. Due to the low percentages of non-classical monocytes in these patients, we decided to focus only on classical and intermediate monocytes. GC boluses induced an increase in CCR2 expression in both classical and intermediate monocytes (1.2 fold and 1.5 fold respectively). Conversely, GC boluses induced a decrease in HLA-DR and CX3CR1 expression in both classical (2.3 fold and 7.9 fold, respectively) and intermediate monocytes (3.3 fold and 4.8 fold, respectively) (**Figure 4**).

### 3.6 Effects of TCZ monotherapy on the expression of CCR2, HLA-DR and CX3CR1 by classical and intermediate monocytes over time

**Figure 5:**
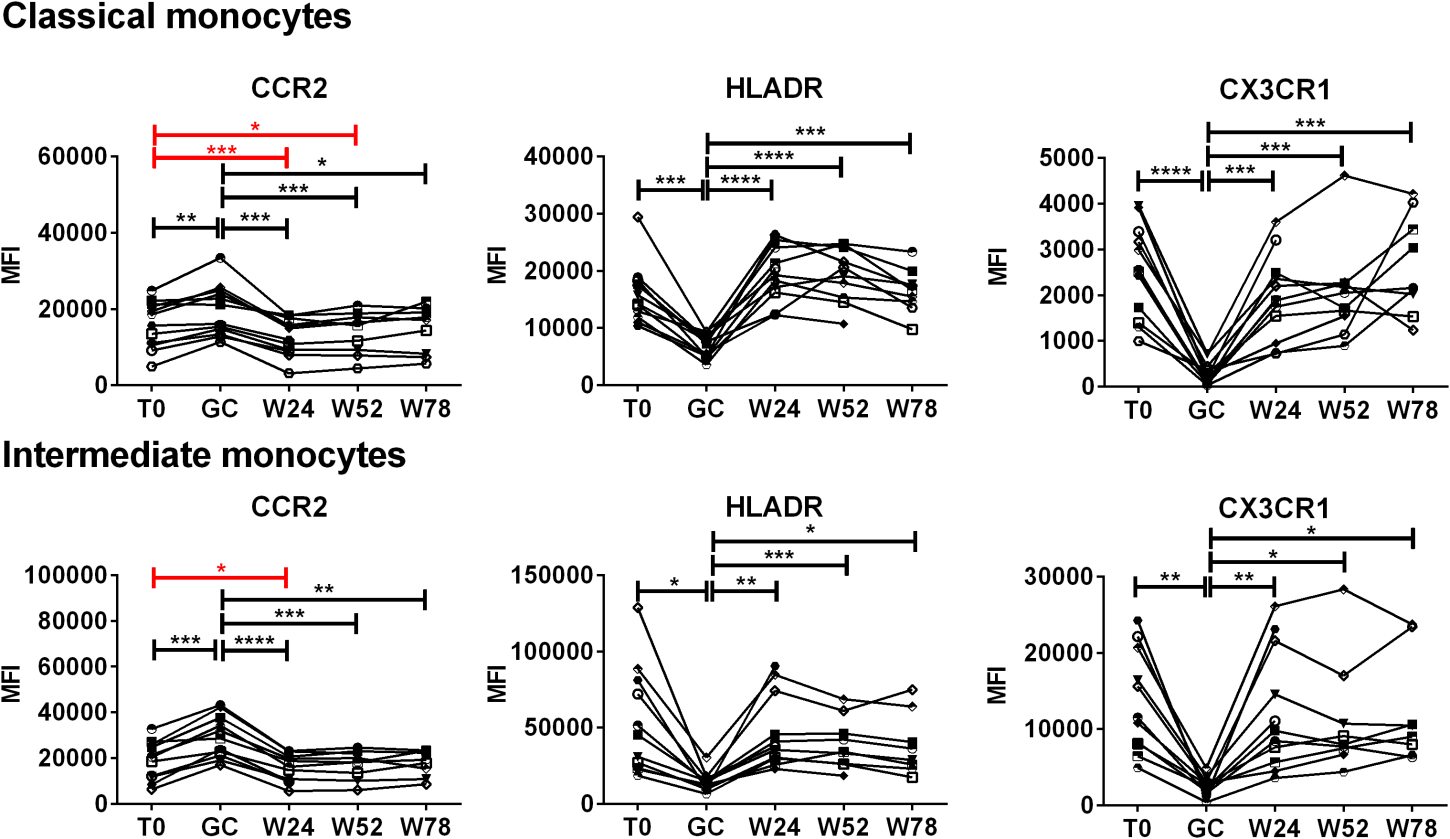
Effects of TCZ monotherapy on the expression of CCR2, HLADR and CX3CR1 by monocyte subsets over time. Monocyte subsets were identified in PBMCs by means of flow cytometry using anti-CD14 and anti-CD16 antibodies. Median fluorescence intensities (MFI) values of CCR2, HLA-DR, CX3CR1 are shown in classical and intermediate monocytes. Data were analyzed at baseline (T0), after 3 days of GC treatment (GC), at 24 (W24) and 52 weeks (W52) of TCZ monotherapy, and at 78 weeks of follow-up (W78). Mixed-effects analysis followed by Tukey’s test correction for multiple comparisons was used to compare MFI data among the time-points. Red lines evidence the statistically significant differences in the expression of CCR2 to the baseline induced by TCZ. Each symbol represents a different patient. P-values<0.05 were considered statistically significant. * = p<0.05; ** = p<0.01; *** = p<0.001; **** = p<0.0001.

To evaluate the effects of treatment with GCs followed by TCZ monotherapy on the expression of CCR2, HLA-DR and CX3CR1 in classical and intermediate monocytes, PBMCs were analyzed longitudinally in each patient (**Figure 5**). During TCZ monotherapy, the expression of CCR2 decreased at both 24 and 52 weeks with respect to the baseline in classical monocytes. The decrease in CCR2 expression was observed also in intermediate monocytes at 24 weeks of TCZ monotherapy. The analysis of the fold changes in CCR2 levels with respect to the baseline evidenced also a decrease in CCR2 at 52 weeks in intermediate monocytes, as well as confirming the above data (**Supplementary Figure S5**). Following the discontinuation of TCZ treatment, at 78 weeks, the expression of CCR2 remained similar to that observed at 52 weeks or increased becoming similar to the baseline. Moreover, concerning HLA-DR and CX3CR1, after the decrease induced by GC boluses, during TCZ monotherapy their expression returned to the expression levels observed at baseline in both classical and intermediate monocytes. The statistically significant increases reported in **Figure 5** are referred to GC treatment.

### 3.7 Changes in CCR2 levels at 24 and 52 weeks in intermediate monocytes as a potential predictor of response to treatment at 78 weeks

**Figure 6:**
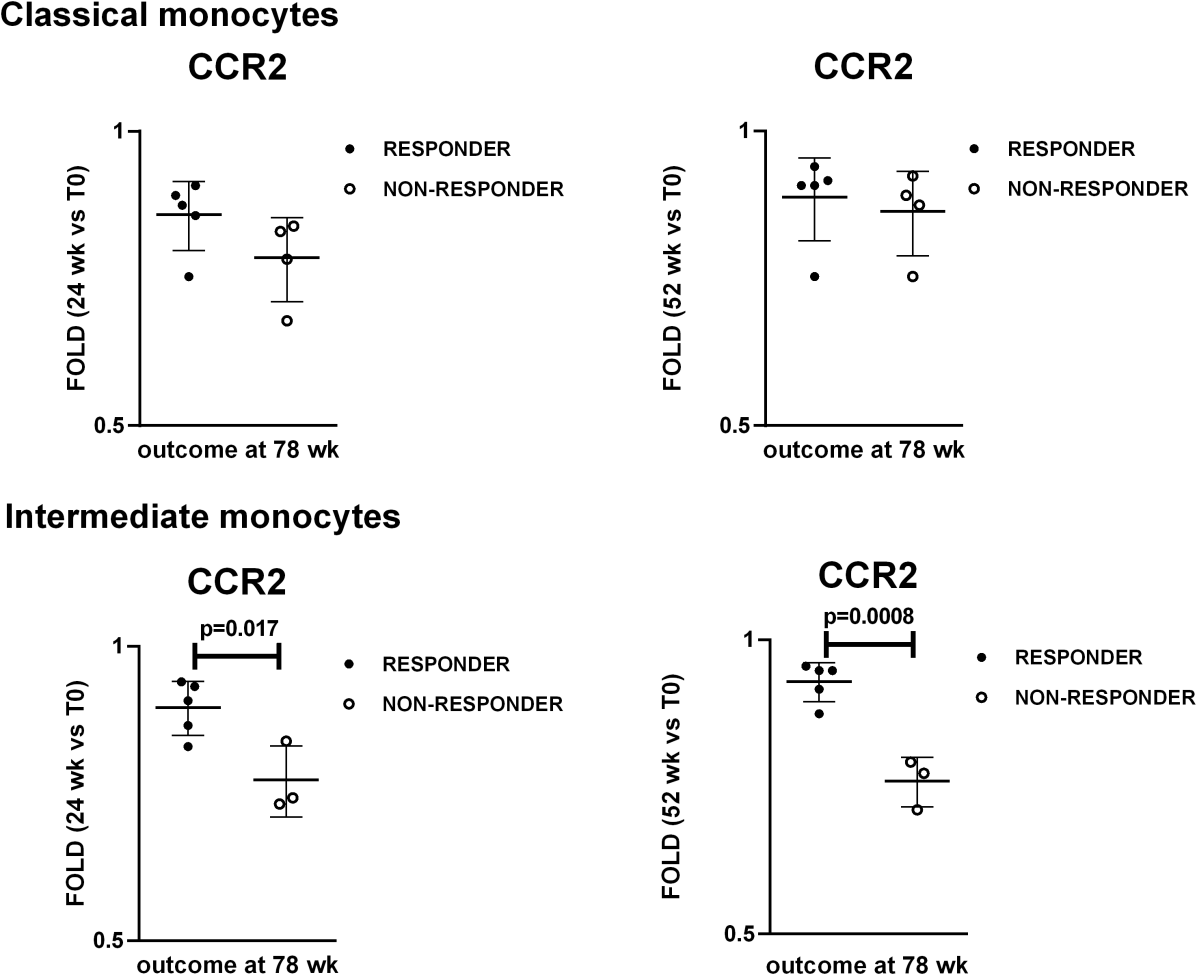
Changes in CCR2 levels at 24 weeks and 52 weeks as potential predictors of response to treatment at 78 weeks. Dot plot visualization of fold-changes in CCR2 expression in classical and intermediate monocytes at 24 wk and 52 wk with respect to the baseline (T0). Full dots represent responder patients whereas empty dots represent non-responder patients. Student’s t-test was used to investigate the differences in fold-changes between responder and non-responder patients. P<0.05 (two-tailed) were considered statistically significant. Horizontal lines represent the median ± interquartile range.

To identify potential “bedside-bench” and “bench-bedside” correlations, we stratified the patients in responders and non-responders to therapy after TCZ discontinuation, and we compared the levels of the investigated markers during the follow up between the two groups of patients. Nine patients completed the follow-up to 78 weeks: 4 patients were classified as non-responders while 5 patients were classified as responders to treatment based on the parameters reported in Materials and Methods paragraph 2.1. PET vascular activity score (PETVAS) during the follow up is shown in **Supplementary Figure S6**. We compared the MFIs of CCR2, HLA-DR and CX3CR1 and fold changes in these MFI in classical and intermediate monocytes, the percentages of classical, intermediate and non-classical monocytes, T, CD4+ T, CD8+ T, B, NK, NKT lymphocytes and increase/decrease in such percentages at 24 and 52 weeks between responders and non-responders at 78 weeks. The analyzed parameters did not differ between responder and non-responder patients, except for fold changes in CCR2 expression levels. The analysis of the fold changes in CCR2 expression levels with respect to the baseline revealed differences between responder and non-responder patients at both 24 and 52 weeks, specifically in intermediate monocytes (**Figure 6**). Patients who showed fold changes in CCR2 expression < 0.815 at 24 weeks and < 0.795 at 52 weeks versus baseline were likely to be non-responders (sensitivity set = 100% with corresponding specificity = 80% at 24 weeks, specificity = 100% at 52 weeks by ROC curve analysis).

## 4 Discussion

The primary objective of this study was to improve understanding of the mechanisms of action of a therapy involving boluses of GCs followed by TCZ monotherapy in patients with LVV (refer to https://clinicaltrials.gov: NCT05394909). LVV is a model of chronic inflammatory disease of vessels. Specifically, we analyzed lymphocyte and monocyte subsets in peripheral blood, as well as the expression of receptors on the surface of monocytes. We focused on the immunological effects of this therapy on immune cell subsets known to be involved in the pathogenesis of GCA. CD4+ T lymphocytes are ones of the main players in the pathogenesis of GCA (14–17). Moreover, B lymphocytes have been reported decreased in peripheral blood (18), and artery tertiary lymphoid organs have been detected in temporal arteries from patients with GCA, suggesting a pathogenic role of B lymphocytes. Additionally, we investigated monocyte subsets and specifically the expression of CCR2 and CX3CR1 due to their documented deregulation in GCA both at tissue level and peripheral blood (19–21). The expression of HLA-DR by monocytes was chosen to evaluate their antigen presentation potential. CCR2 is the receptor for the chemokine CCL2, which primarily mediates monocyte chemotaxis, while CX3CR1 binds the chemokine CX3CL1 (fractalkine), regulating leukocyte migration and adhesion. We also assessed HLA-DR expression, an MHC class II cell surface receptor, to evaluate antigen presentation capacity.

GCs exert both genomic and non-genomic effects. Genomic effects occur through cytosolic GC receptors that, upon GC binding, translocate to the nucleus and regulate gene transcription via glucocorticoid response elements. Non-genomic effects derive from: (1) interactions with cellular membranes; (2) membrane-bound GC receptors; and (3) cytosolic GC receptors (22). The GC boluses used in this study, as well as pulse GC therapy (≥ 250 mg/day prednisone-equivalent for 1–5 days), are expected to saturate all GC receptors, ensuring full genomic effects. Additionally, they may exert non-genomic effects and pharmacodynamics effects (23).

Such high doses are clinically used for the initial treatment of acute or life-threatening exacerbations of rheumatic diseases. In the TOPAZIO study, we selected GC boluses over oral GCs to achieve faster effects, including non-genomic actions. Since patients were in the active disease phase, our goal was to suppress GCA clinical manifestations and prevent severe ischemic complications, particularly blindness. While TCZ is usually combined with GCs, in this study it was used as monotherapy. We designed the TOPAZIO study based on the positive results of the GUSTO trial, which also evaluated TCZ monotherapy after GC boluses (11). Additionally, we considered the positive GC-sparing effects of the double-blind, placebo-controlled, randomized prospective trial on cranial GCA treatment using pulse GC induction therapy (24).

GC boluses increased the relative ratio between B and T lymphocytes. It is well established that GCs can influence the absolute numbers of lymphocytes and monocytes (25). Lymphopenia with reductions in CD4+ and CD8+ T lymphocytes, has been observed in patients receiving chronic methylprednisolone for kidney transplantation (26). Regarding B lymphocytes, GCs can either inhibit their activity by inducing apoptosis or stimulate their proliferation and antibody production, depending on specific physiological contexts (27). We speculate that the increase in B lymphocyte percentages and the decrease in T lymphocyte percentages observed after GC boluses likely result from T lymphocyte depletion.

Based on the expression of CD14 and CD16, monocytes are classified into three phenotypically and functionally distinct groups: classical, intermediate and non-classical. Classical monocytes, the most abundant in peripheral blood, are involved in phagocytosis, innate immune responses, and migration. Intermediate monocytes are characterized by pro-inflammatory activities, including cytokine production and increased antigen presentation capacity. Non-classical monocytes participate in antiviral responses, phagocytosis mediated by complement and IgG constant fragment receptors, and the clearance of damaged cells and debris from the vascular system (28). The increase in classical monocytes and the reduction in intermediate and non-classical monocytes observed after GC boluses could contribute to the anti-inflammatory effects of GCs.

The impact of GC boluses on monocyte subsets that we observed aligns with findings reported by other researchers. In a cohort of multiple sclerosis patients, high-dose GC treatment induced an increase in classical monocytes and a decrease in intermediate monocytes (29). Similarly, in healthy donors, high-dose GC infusion resulted in a depletion of intermediate monocytes. Additionally, *in vitro* treatment of PBMCs with methylprednisolone induced a reduction of intermediate monocytes through apoptosis (30). In patients with immune thrombocytopenia, 2 weeks of prednisolone treatment reduced the percentages of intermediate monocytes and increased the expression of anti-inflammatory markers on monocytes (31). Lastly, in animal models of osteoporosis, exogenous GC administration stimulated the expansion of classical monocytes in the bone marrow, with potential for osteoclast differentiation (32).

Following GC administration, patients showed a decrease in CX3CR1 and HLA-DR expression, along with an increase in CCR2 expression across all monocyte subsets. The reduced expression of CX3CR1 aligns with findings by Yannick van Sleen et al., though the increased CCR2 expression differs from their results (20). It is important to note that differences in GC doses and schedules between the two studies may account for this discrepancy.

Decreased CX3CR1 and HLA-DR expression could contribute to the anti-inflammatory effects of GCs, by reducing leukocyte chemotaxis, cell adhesion, and antigen presentation. This is supported by literature indicating that GCs down-regulate CX3CR1 and HLA-DR. Murine monocytes treated with dexamethasone for 48 hours showed down-regulation of CX3CR1, similar to human monocytes treated with GCs (33). Additionally, a reduction in HLA-DR was observed on classical and non-classical monocytes from healthy donors treated with hydrocortisone (34).

The increased expression of CCR2 following GC boluses was unexpected, as it could potentially increase monocyte migration to tissues, which seems at odds with the anti-inflammatory effects of GCs. However, literature reports increased CCR2 expression and monocyte chemotaxis induced by GCs (35, 36). CCR2 plays a dual role, exhibiting both pro- and anti-inflammatory activities. At low doses of CCL2, the receptor’s ligand, CCR2 can inhibit T lymphocyte trafficking and differentiation (37). Additionally, CCR2-expressing monocytes/macrophages in the tumor microenvironment can be strongly immunosuppressive (38).

Following TCZ monotherapy, the only observed effect was a reduction in CCR2 levels on classical and intermediate monocytes. Since IL-6 and CCR2 can mutually induce each other (39), this reduction is rationale. It is known that monocytes express on the plasma membrane IL-6R. They are one of the main target of IL-6 and therefore are the cells that should be most affected by IL-6 signaling inhibition by TCZ. The fact that the percentages of monocyte subsets were not affected by TCZ monotherapy can be explained hypothesizing that IL-6 signaling does not regulate monocyte differentiation in peripheral blood. On the other hand, we cannot rule out that the absolute number of the classical, intermediate and non-classical monocytes were affected. Since post-thawed PBMCs were analyzed instead of fresh whole blood, we couldn’t determine the absolute number of cells.

The lack of significant differences in the percentages of CD19+ B, NK, NKT, T, CD4+ T, and CD8+ T lymphocytes during and after TCZ monotherapy compared to baseline can be explained by the fact that these cells did not express or express at low levels plasma membrane IL-6R. Therefore, classical IL-6 signaling is unlikely in these immune cells. Any effects of TCZ on these cells, if present, are expected to result primarily from the inhibition of IL-6 trans-signaling. Similarly to our data, Jouve et al. reported that kidney transplant candidates undergoing 6 months of intravenous TCZ treatment at 8 mg/kg every 4 weeks had no changes in the percentages of CD4+ T, CD8+ T, CD19+ B and CD56+ NK cells (40). Moreover, as stated previously, due to the study design we cannot rule out that the absolute number of the lymphocyte subsets were affected. It has been reported that patients with GCA have increased frequencies of Th1 and Th17 cells. GCs can inhibit Th17 responses in the inflamed arteries and in the peripheral blood, whereas Th1 responses are spared and can be involved in refractory disease (41). We found that TCZ did not affect the overall percentages of CD4+ T lymphocytes, but Th cell subset analysis was not performed. Future studies are needed to explore whether TCZ differentially affects Th subsets.

To identify potential translational impact, we stratified the patients in responders and non-responders to therapy after TCZ discontinuation, and we compared the levels of the investigated markers during the follow up between the two groups of patients. Only the analysis of the fold changes in CCR2 expression with respect to the baseline revealed differences between responder and non-responder patients, specifically in intermediate monocytes. Patients with a greater reduction in CCR2 levels on intermediate monocytes showed signs of disease activity after TCZ discontinuation at 78 weeks. We thus speculate that the longitudinal analysis of CCR2 expression by intermediate monocytes might be of help to identify patients at higher risk of flare. Moreover, these findings suggest that low CCR2 expression by monocytes might be unfavorable in this disease context.

Strength of this study is the use of biological samples from patients treated with TCZ to determine the drug’s effects, rather than relying on *in vitro* PBMC treatments. Additionally, this is the first study to analyze the effects of GC boluses followed by TCZ monotherapy on monocyte and lymphocyte subsets in patients with LVV. However, the study has some limitations, including the small sample size and the lack of longitudinal samples from all patients up to 78 weeks. Besides, additional time points could have helped determine when GCs stop exerting their immunomodulatory effects and when TCZ starts having biological impact. Furthermore, since post-thawed PBMCs were analyzed instead of fresh whole blood, we couldn’t determine the absolute number of cells. Therefore, the observed increase in the percentages of a cell subset could result either from an increase in the absolute number of that subset or from the depletion of another subset, which would affect the relative percentages. On the other way round, in case of unchanged percentages, it cannot be ruled out changes in the cell absolute numbers. However, the use of post-thawed PBMCs allowed us to analyze all the longitudinal samples from each patients in the same experiment lowering technical variability and strengthening the comparisons. Future studies on larger, independent cohort of patients followed longitudinally at multiple time points would be needed to further confirm the results. In addition, it would be worthwhile to perform the analysis of both freshly collected and post-thawed PBMCs. Finally, future studies should explore whether TCZ can differentially affects Th subsets.

Overall, GC boluses modified the relative percentages of lymphocyte and monocyte subsets and affected the expression levels of CCR2, CX3CR1 and HLA-DR on monocytes. TCZ monotherapy had limited effects. Monitoring CCR2 expression by intermediate monocytes might have a prognostic value in LVV.

## Data Availability

The original contributions presented in the study are publicly available. This data can be found here: http://flowrepository.org/experiments/9290; FlowRepository.org ID: FR-FCM-Z92A.
