# Supplementary material for: Glucocorticoid boluses followed by tocilizumab in giant cell arteritis patients: effects on peripheral blood monocytes and lymphocytes
Source: Front Immunol. 2025 Mar 27;16:1516008. doi: 10.3389/fimmu.2025.1516008 (PMC11983418; doi:10.3389/fimmu.2025.1516008)
Supplement: Supplementary file 1 [file DataSheet1.docx]

Supplementary Material

# Supplementary Tables

# Supplementary Table S1. Clinical characteristics of patients at baseline.

| **Variables** | **Values** | **Reference Values** |
| --- | --- | --- |
| Males | 4/15 | Not applicable |
| Females | 11/15 | Not applicable |
| VES (mm/h) | 30 (12.5-56.5)* | 2-37 |
| PCR (mg/l) | 1.22 (0.51-3.34)* | <0.5 |
| Polymyalgia rheumatica | 4/15 | Not applicable |
| PET/CT | Grade 2 and 3 | Not applicable |

^*^ median (25^th^-75^th^ percentile)

# Supplementary Figures

**
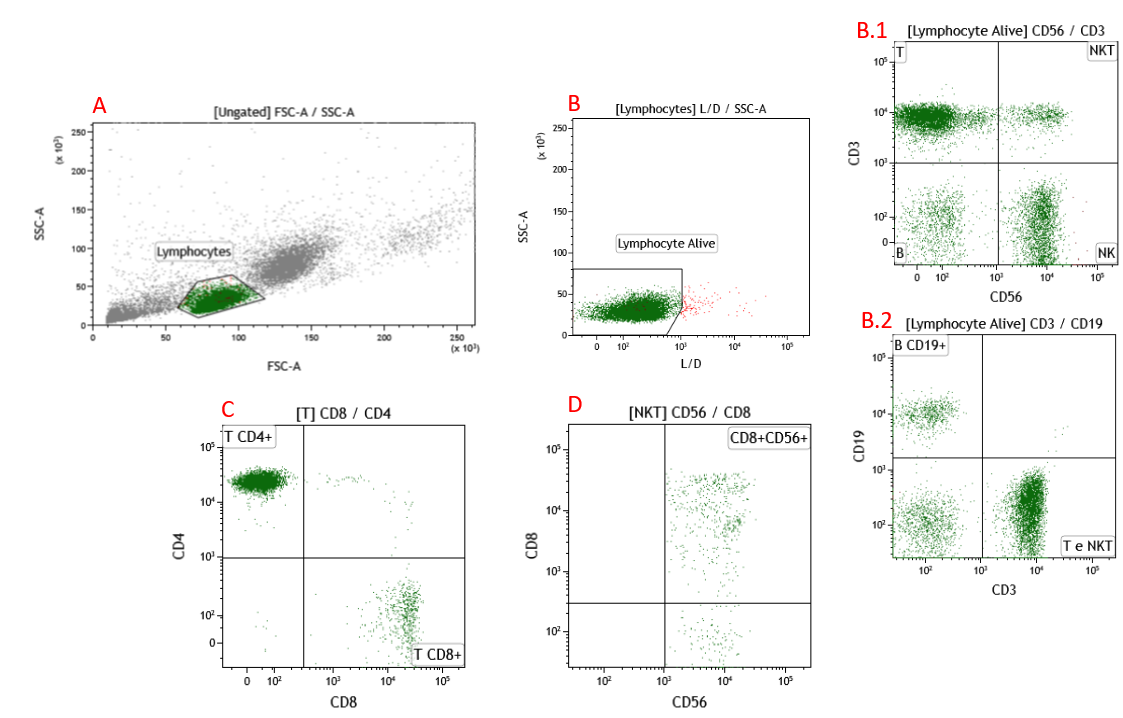
**

**Supplementary Figure S1.** Gating strategy for lymphocyte profiling. A. Selection of lymphocytes based on forward scatter (FSC) and side scatter (SSC). B. Selection of lymphocytes alive based on Live/Dead (L/D) staining. B.1 Identification of CD3+CD56- T cells, CD3+CD56+ NKT cells and CD56+CD3- NK cells. B.2 Identification of CD19+CD3- B cells C. Identification of CD4+T cells and CD8+ T cells. D. Identification of CD8+CD56+ NKT cells and CD8-CD56+ NKT cells.

**
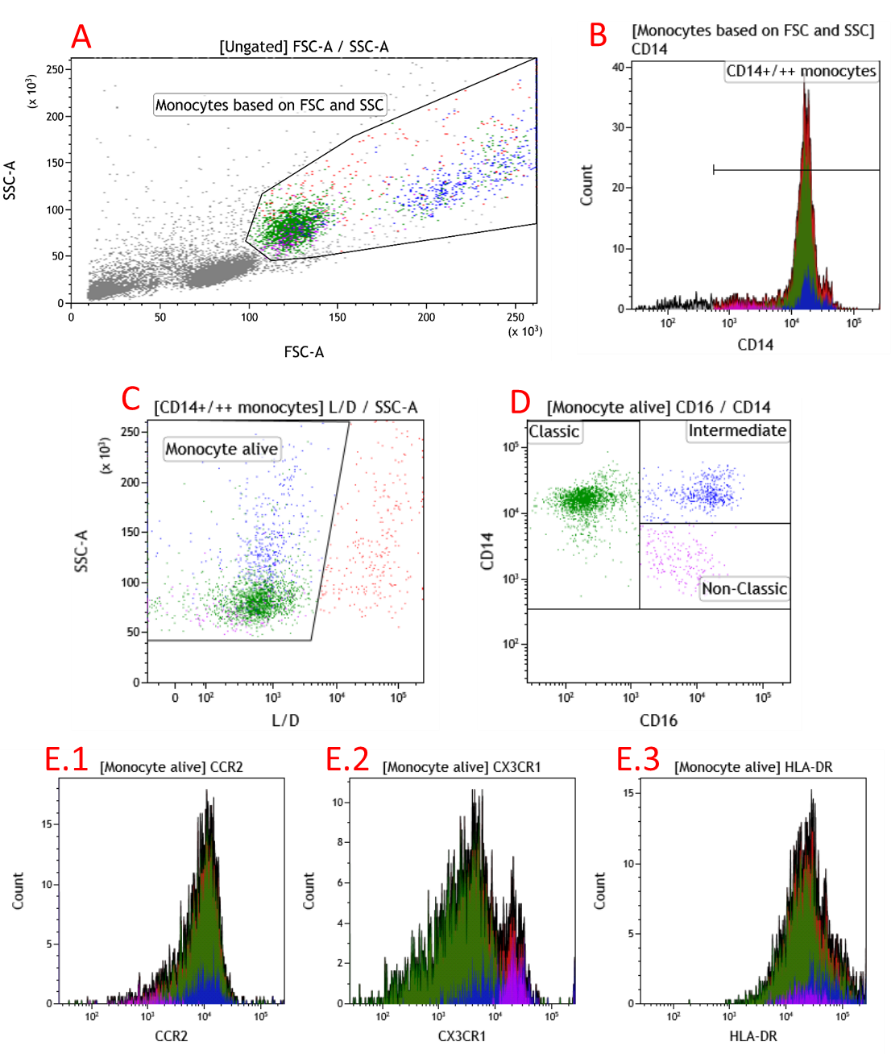
**

**Supplementary Figure S2.** Gating strategy for monocyte profiling and analysis of the expression of monocyte markers CX3CR1, HLADR, CCR2. A. Selection of monocytes based on forward scatter (FSC) and side scatter (SSC). B. Selection of CD14+ monocytes. C. Selection of monocytes alive based on Live/Dead (L/D) staining. D. Identification of CD14+CD16- classical monocytes, CD14+CD16+ intermediate monocytes and CD14lowCD16+ non-classical monocytes. Evaluation of the MFI of CCR2 (E.1), CX3CR1 (E.2) and HLA-DR (E.3) to analyze their expression on monocytes.





**Supplementary Figure S3.** Effects of TCZ monotherapy on lymphocyte subsets over time. Lymphocyte subsets were identified in PBMCs by means of flow cytometry using the following surface markers: CD3, CD4, CD8, CD19, and CD56. Percentages were calculated in the lymphocyte gate defined on forward and side scatter of the cells. Data were analyzed at baseline (T0), after 3 days of GC treatment (GC), at 24 (W24) and 52 weeks (W52) of TCZ monotherapy, and at 78 weeks of follow-up (W78). Mixed-effects analysis followed by Tukey’s test correction for multiple comparisons was used to compare data among the time-points. Only patients with at least one time-point on TCZ treatment were considered for the analysis. Each symbol represents a different patient. P-values < 0.05 were considered statistically significant.

**

**

**Supplementary Figure S4.** Effects of TCZ on monocyte subsets over time. Monocyte subsets were identified in PBMCs by means of flow cytometry using anti-CD14 and anti-CD16 antibodies. Data are expressed as fold-changes of the percentages of classical, intermediate and non-classical monocytes after 3 days of GC treatment (GC), at 24 (W24) and 52 weeks (W52) of TCZ monotherapy, and at 78 weeks of follow-up (W78) with respect to the baseline (T0). Mixed-effects analysis followed by Tukey’s test correction for multiple comparisons were used to compare the fold changes, among the time-points. Only patients with at least one time-point on TCZ treatment were considered for the analysis. Each symbol represents a different patient. Dotted blue lines indicate the reference value of baseline. P-values < 0.05 were considered statistically significant.

**

**

**Supplementary Figure S5.** Effects of TCZ on the expression of CCR2, HLADR and CX3CR1 by classical and intermediate monocytes over time. Monocyte subsets were identified in PBMCs by means of flow cytometry using anti-CD14 and anti-CD16 antibodies. Median fluorescence intensities (MFI) values of CCR2, HLADR, CX3CR1 were determined in classical and intermediate monocytes. Fold change of the MFI of CCR2, HLADR and CX3CR1 after 3 days of GC treatment (GC), at 24 (W24) and 52 weeks (W52) of TCZ monotherapy, and at 78 weeks of follow-up (W78) with respect to the baseline (T0) are shown. Mixed-effects analysis followed by Tukey’s test correction for multiple comparisons were used to compare the fold changes, among the time-points. Only patients with at least one time-point on TCZ treatment were considered for the analysis. Red lines evidence the statistically significant fold change in the expression of CCR2 with respect to the baseline induced by TCZ. Dotted blue lines indicate the reference value of baseline. Each symbol represents a different patient. P-values < 0.05 were considered statistically significant.

**
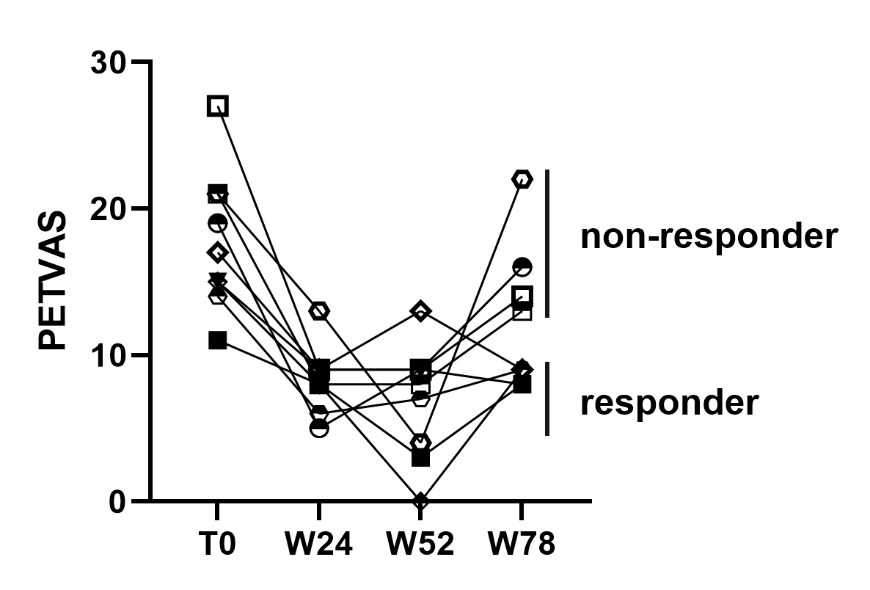
**

**Supplementary Figure S6.** PET vascular activity score (PETVAS) during the follow-up.
